# Encapsulation of Bioactive Compounds from Germinated Mung Bean by Freeze-Drying, Release Kinetics, and Storage Stability

**DOI:** 10.3390/foods13010100

**Published:** 2023-12-27

**Authors:** Anh Thuy Vu, Tuyen Chan Kha, Huan Tai Phan

**Affiliations:** Faculty of Chemical Engineering and Food Technology, Nong Lam University, Ho Chi Minh City 700000, Vietnam; vuthuyanh@hcmuaf.edu.vn (A.T.V.); pthuan@hcmuaf.edu.vn (H.T.P.)

**Keywords:** encapsulation, freeze-drying, GABA, mechanism kinetics, polyphenol, release kinetics, storage stability

## Abstract

This research explores the application of germinated mung bean extract, rich in GABA (Gamma-aminobutyric acid) and polyphenols, in enhancing human health. Recognizing the instability of these bioactive compounds in environmental conditions, encapsulation emerges as a pivotal technique to broaden their applications in food and pharmaceuticals. Utilizing response surface methodology and Box–Behnken design, the freeze-drying formulation for encapsulating the aqueous extract was optimized. Second-order polynomial models were developed, exhibiting statistical adequacy in predicting key variables such as encapsulation efficiency for GABA (EE-GABA) and total polyphenol content (EE-TPC), as well as encapsulation yield for GABA (EY-GABA) and total polyphenol content (EY-TPC). The established optimal formulation was validated, resulting in predicted values for EE-GABA, EE-TPC, EY-GABA, and EY-TPC. The release kinetics of encapsulated particles were investigated, highlighting the suitability of the Korsmeyer–Peppas and Higuchi models. Assessing the stability of the encapsulated powder under varying temperatures and humidities revealed degradation rates, half-life, and activation energy, with moisture equilibrium established at 4.70%, indicative of long-term stability. In conclusion, the encapsulated germinated mung bean powder demonstrates high stability, making it a promising candidate for integration into food products and functional ingredients.

## 1. Introduction

Extract rich in GABA (Gamma-aminobutyric acid) and polyphenols from germinated mung beans plays a crucial role in human health. However, these compounds are often unstable and highly susceptible to degradation under environmental conditions such as temperature, light, air, and humidity. Consequently, their application in food and pharmaceuticals is limited. To overcome this limitation, one effective technique is to encapsulate these bioactive compounds to prevent exposure to environmental conditions, while enabling diverse applications in various fields.

One of the primary purposes of encapsulation techniques in food is that the core material, sensitive to environmental conditions such as temperature, humidity, light, and air, can be protected. The reactions of bioactive compounds in the mixture can be isolated, and the release rate of the core material from the wall material can be controlled under appropriate conditions (temperature, radiation, pH, or permeability) at a specific time [1]. The number of encapsulated foods or pharmaceuticals has significantly increased in the past decade. Recently, various core materials (e.g., GABA and polyphenols) have been encapsulated by different wall materials and produced through various encapsulation techniques based on their specific applications. However, there are few reports on the encapsulation of GABA using various wall materials and drying techniques. For instance, GABA has been encapsulated through spray drying, achieving an encapsulation efficiency higher than 80% by using inulin, dextran, and maltodextrin as wall materials [2]. Alternatively, freeze-drying has been employed, achieving an encapsulation efficiency of 90% with wall materials such as dextran and whey protein [3]. Furthermore, Rout et al. [4] reported a high encapsulation efficiency of up to 96% for polyphenols in oregano leaf extract using freeze-drying and wall materials comprising maltodextrin and gum arabic. To the best of our knowledge, no study has investigated the encapsulation of GABA and TPC using freeze-drying with gum arabic (GA) and whey protein (WP) as wall materials, especially in the context of GABA and polyphenols in germinated mung bean extract.

One of the prerequisite factors determining the success of encapsulation techniques is the selection of a suitable wall material. As each type of wall material possesses distinct characteristics, with advantages and disadvantages in terms of encapsulating and stabilizing the core material, it is essential to investigate the combination of various wall materials. Among different wall materials derived from carbohydrates, proteins, and lipids, GA stands out due to its low viscosity, high water solubility, and emulsifying properties. On the other hand, WP excels in its superior encapsulation ability for amphophilic (hydrophilic and hydrophobic) compounds and offers nutritional benefits. These two, GA and WP, are widely recognized as the most popular wall materials for encapsulating hydrophobic active compounds [5]. According to Sun-Waterhouse et al. [6], different wall materials exhibit diverse physical and chemical properties, necessitating a synergy among these materials to effectively protect and control the bioactive compounds. Klein et al. [7] reported that wall materials containing GA and WP could enhance the stability of emulsions containing bioactive compounds, resisting a significant increase in droplet size, resulting in high encapsulation efficiency. Similarly, our preliminary results indicated that a combination of GA and WP showed higher values of encapsulation efficiency and encapsulation yield compared to using either one alone.

Encapsulation techniques involve three main stages: selecting a suitable formulation, choosing an encapsulation method, and determining the release rate and mechanism. Although the release of bioactive compounds from encapsulated particles is the final step of encapsulation, the release rate and mechanism must be considered from the outset, depending on the encapsulation purpose. This is because the release characteristics are significantly influenced by the choice of wall material and encapsulation method. Controlling the release of the core material is one of the most crucial features of encapsulated particles. Therefore, understanding and determining the release characteristics of encapsulated bioactive compounds at the desired time and environment are essential. The release rate of encapsulated particles is experimentally determined under the actual conditions to which they will be applied. The release rate of bioactive compounds from encapsulated particles can follow zero-order or first-order kinetics or other mathematical models. Studying the kinetics of the release rate to understand release characteristics is crucial. The release rate depends on the properties of the wall material, the characteristics of the encapsulated particles, and the actual environmental conditions [8].

In addition to the inherent quality changes during processing, the quality of food products may undergo alterations during storage and distribution. Investigating the impact of storage conditions on the quality of encapsulated powders becomes imperative due to the continuous occurrence of deteriorative reactions affecting their color and nutrient properties over time. Factors such as light, temperature, and oxygen are recognized as influential in determining the quality of powders. Foods containing high unsaturated fatty acids and carotenoids are particularly susceptible to accelerated deterioration when exposed to inappropriate conditions, such as elevated temperatures. Furthermore, degradation reactions can be initiated by enzymatic activity, lipid oxidation, and non-enzymic browning, which often lead to undesirable changes in color, flavor, and nutritional content [9]. Thus, comprehending the causes of deterioration and identifying suitable storage conditions for food products is crucial. However, there is a lack of reported information regarding the stability of the encapsulated germinated mung bean extract powder product during prolonged storage, necessitating further investigation.

The objectives of this study were (i) to determine the optimal wall formulation for encapsulating the germinated mung bean extract using the freeze-drying method, (ii) to identify the model and mechanism of releasing GABA and polyphenols from the encapsulated powder under different environments, and (ii) to determine the kinetic parameters of GABA and polyphenol loss during the storage.

## 2. Materials and Methods

### 2.1. Chemicals and Materials

Alcalase 2.5 L PF with an activity of 2.5 AU-A/g was procured for this research from Novozymes (Bagsværd, Denmark). Trypsin (an activity of 2500–6000 BAEE U/mL) and pepsin (an activity of 0.7 FIP-U/mg) were purchased from Sigma-Aldrich Pty Ltd. (St. Louis, MI, USA). All the chemicals and reagents used in the study, such as ethanol, sodium hypochlorite, sodium carbonate anhydrous, sodium phosphate, sodium dodecyl sulfate, hydrochloric acid, phenol, boric acid, and Folin–Ciocalteu, were of analytical grade and obtained from Merck (Darmstadt, Germany. Standard substances, specifically GABA and gallic acid, were sourced from Sigma Aldrich (St. Louis, MI, USA).

Mung beans, specifically of the DX208 variety, were procured from the Southern Seed Corporation in Ho Chi Minh City, Vietnam. To ensure quality, the beans underwent a selection process involving the removal of broken, flat beans, and impurities.

Gum arabic (GA, Alland & Robert, Paris, France) and whey protein (WP, Hilmar Ingredients, Hilmar, CA, USA) were utilized as wall materials.

### 2.2. Preparation of the Core Material

The initial step involved soaking the beans in water at a ratio of 1:5 (*w*/*w*) at 30 ± 1 °C for 8 h, following the procedure outlined by Vu et al. [10]. Post-soaking, the beans were thoroughly washed and arranged on a stainless-steel tray in a 1 cm layer. This tray was then placed in an incubator set at 35 ± 1 °C with 90% humidity for 12 h, in accordance with the method reported by Vu et al. [11]. Upon completion of the incubation period, the germinated mung beans were removed, washed with distilled water to eliminate mucilaginous substances from the surface, allowed to drain for 10 min, and subsequently subjected to extraction with the assistance of alcalase. Optimal extractions, including temperature of 53 ± 1 °C, a time of 62 min, and an enzyme concentration of 2.2%, were employed according to the method described by Vu et al. [12]. The resultant extract, characterized by a GABA content of 57.73 ± 1.28 mg/g, dry weight, dw) and total polyphenols (TPC) measuring 6.81 ± 0.42 mg GAE/g, dw, was used as the core material.

### 2.3. Preparation of the Extract Microparticles

The extracted fraction was blended with different ratios of wall materials, including GA and WP. To form the wall solution, the wall materials were mixed with water at a ratio of 3:10 (*w*/*v*) and stirred on a magnetic stirrer at 60 °C for 30 min. Subsequently, the extracted fraction was combined with the wall solution at a ratio of 1:5 (*v*/*v*). The mixture was homogenized using a high-speed homogenizer (T 25 digital ULTRA-TURRAX^®^, IKA, Staufen, Germany) at 10,000 rpm for 5 min. The resulting solution was weighed, poured into petri dishes, and frozen at −18 °C. After complete freezing, the samples were lyophilized at −51 °C for 48 h using a freeze-dryer (BK-FD10S, Biobase, Jinan, China) [13]. Following the lyophilization process, the samples were finely ground into powder using a laboratory blender (Lock&Lock EJM462, Ho Chi Minh City, Vietnam) and vacuum-sealed for the analysis of powder characteristics (moisture content, encapsulation efficiency, encapsulation yield, and powder yield).

### 2.4. Experimental Design

#### 2.4.1. Optimization of Formulation

After selecting suitable wall materials, determining the optimal ratio of the extract to wall material, and identifying the appropriate coating material concentration from our preliminary experiments, experiments to optimize the encapsulation formula for encapsulating bioactive compounds from germinated mung bean extract were carried out. An experimental matrix was constructed, and the experiments were arranged using the Box–Behnken design to identify the most influential factors in the encapsulation process of germinated mung bean extract. The selected factors for investigation included the ratio between two wall materials, including GA and WP (X_1_, *v*/*v*), the ratio of the extract to wall material (X_2_, *v*/*v*), and the wall material concentration (X_3_, %). The independent variables were coded and uncoded in the experimental matrix, as presented in Table 1.

#### 2.4.2. Modeling the Release Rate under Different Conditions

A study on the release of GABA and polyphenols in encapsulated mung bean extract powder was conducted under three different temperatures (25, 37, and 45 °C), three pH conditions (2, 5, and 8), and three environments (water, simulated gastric fluid, SGF, and simulated intestinal fluid, SIF). The chosen temperatures provided a preliminary investigation to gain an overall understanding of the variations in GABA and polyphenols release under different conditions. Specifically, 25 °C represented an ideal environmental temperature, and 45 °C simulated harsh summer conditions [4] or an appropriate temperature for dissolving raw materials before further processing steps. Combining these temperatures with pH levels of 2, 5, and 8 was selected to understand the storage conditions of these bioactive compounds when incorporated into foods with low, medium, and high pH levels. The temperature of 37 °C and three pH levels were chosen based on digestion conditions in the human body [4].

Two milliliters from each previously prepared buffer solution were placed into separate test tubes, and 0.2 g of encapsulated mung bean extract powder was added. The samples were stored at different temperatures of 25, 37, and 45 °C. The release amount of GABA and TPC were analyzed at time intervals of 5, 10, 30, 60, and 120 min.

SGF was prepared by adjusting a 0.1 M sodium phosphate buffer solution to the study pH levels using 2 N HCl, followed by adding approximately 2.0 g of pepsin before analysis. SIF was prepared by dissolving 2.0 g of trypsin and sodium dodecyl sulfate in a 0.1 M phosphate buffer solution, then adjusting the pH to the desired levels as above using 2 N NaOH [14].

The kinetics of GABA and TPC release under different conditions were calculated using various models: zero-order, first-order, Higuchi model, and Korsmeyer–Peppas [4].

#### 2.4.3. The Effect of Storage Conditions

The optimal encapsulated extract powder (2 g) was vacuum-sealed and stored at different temperatures, including 30, 40, and 50 °C for different times (0, 4, 10, 21, 25, and 30 days). Samples of the encapsulated powder were periodically taken for analysis to assess the changes in GABA and TPC under different storage conditions.

To determine the impact of storage conditions on the variation in the levels of bioactive compounds, dynamic parameters such as reaction rate constants (k), activation energy (Ea), and the storage time (t) of the encapsulated powder were calculated from the collected data.

#### 2.4.4. The Effect of Air Relative Humidity

The optimal encapsulated extract powders (2 g) were weighed into aluminum cups and then placed into desiccators containing saturated solutions of sodium hydroxide (NaOH), potassium acetate (CH_3_COOK), magnesium nitrate (Mg(NO_3_)_2_), sodium chloride (NaCl), and potassium chloride (KCl), creating a range of relative humidity from 8 to 84% [15]. The relative humidity levels were monitored and confirmed using a water activity measurement device. The desiccators containing the encapsulated powder samples and saturated salts were sealed and stored at ambient temperature (25–30 °C). Once equilibrium was reached, the final moisture content was determined using the air-drying method at 105 °C until a constant weight was achieved. After collecting the data, equilibrium moisture content calculations were performed based on the appropriateness of the BET and GAB models.

### 2.5. Analytical Methods

#### 2.5.1. Moisture Content Determination

The analyzed samples were dried to a constant weight at a temperature of 105 °C. The sample (1 g) was placed on a sample dish and subjected to drying at 105 °C. After achieving a constant weight, the final moisture content was determined.

#### 2.5.2. Water Activity Determination

The water activity of the samples was measured by weighing 1 g of the sample into a cup and utilizing the Aqualab water activity (Aqualab Pre, Decagon, Pullman, WA, USA) measurement device for precise assessment.

#### 2.5.3. GABA Content Determination

The GABA content was determined using the method described by Watcha-rarparpaiboon et al. [16], with slight modifications. Briefly, about 0.5 g of encapsulated powder was weighed into a container and dissolved in 10 mL of distilled water. The mixture was shaken for 1 min, then centrifuged at 5200 rpm for 5 min at 4 °C. Approximately 0.6 mL of the extracted solution was added to a test tube, followed by the addition of 0.4 mL of a 0.4 M borate buffer mixture and 2 mL of 6% phenol reagent. Subsequently, 2 mL of 8% NaOCl reagent was added, and the tube was vigorously shaken for 5 min. The resulting mixture was incubated at 90 °C for 10 min, cooled with ice water for 10 min, and the absorbance was measured at a wavelength of 630 nm using a spectrophotometer (Spectro UV 11, Duisburg, Germany). GABA was used as the standard, and the results were expressed in mg of GABA per gram of dry weight (mg/g dw) of the sample.

#### 2.5.4. Total Polyphenol Content Determination

The method for determining total polyphenol content followed the protocol outlined by Złotek et al. [17]. In brief, 0.5 mL of the extracted sample, which was prepared as in Section 2.5.3, was pipetted into a capped test tube, followed by the addition of 2 mL of distilled water. Subsequently, 500 µL of Folin–Ciocalteu reagent (in a 1:10 reagent-to-water ratio) and 2 mL of 7.0% Na_2_CO_3_ were added. The mixture was thoroughly shaken and allowed to stand for 8 min at room temperature. The mixture was left undisturbed for 1 h at room temperature. A parallel analysis was conducted with a blank sample containing the extraction solvent. The total polyphenol content was measured at a wavelength of 765 nm. The total polyphenol content was determined based on the dry weight of the material, using gallic acid as the standard, and the results were expressed in mg GAE per gram of dry weight (mg GAE/g dw) of the sample.

#### 2.5.5. Surface Content of GABA and TPC Determination

To determine the surface content of GABA and TPC [18], about 1 g of encapsulated powder was weighed and dissolved with 5 mL of ethanol. The mixture was shaken for 1 min, and the analysis of GABA (Section 2.5.3) and TPC (Section 2.5.4) was then carried out following the procedure outlined above.

#### 2.5.6. Encapsulation Efficiency and Encapsulation Yield

Encapsulation efficiency (EE) and encapsulation yield (EY) are calculated using the following formulas [13,19]:

$$EE\;(\%)=\frac{m_{p}-m_{s}}{m_{p}}\times 100$$

$$EY\left(\%\right)=\frac{m_{p}}{m_{i}}\times 100$$

Here, EE and EY represent the encapsulation efficiency and encapsulation yield for GABA or TPC, respectively; m_p_ is the mass of GABA or TPC in the encapsulated powder; m_s_ is the mass of GABA or TPC on the surface of the encapsulated powder; and m_i_ is the mass of GABA or TPC in the pre-drying solution.

#### 2.5.7. Morphological Analysis

The morphological characteristics of the encapsulated microparticles were examined using a scanning electron microscope (Hitachi Fe-SEM s4800, Tokyo, Japan). A small amount of the microparticles was placed on a sample holder. Images were captured at 10.0 kV with different magnifications of 100, 500, and 1000 times.

#### 2.5.8. Particle Size Distribution Analysis

The particle size and size distribution of the encapsulated powder were determined using a laser diffraction particle size analyzer, LA-350 Horiba (Kyoto, Japan). Initially, the encapsulated powder was diluted with distilled water to ensure a suitable range of dispersion, typically between 10% and 20%, before analysis. The refractive index of the emulsion droplets was 1.670, and that of the dispersing medium (water) was 1.33.

#### 2.5.9. Kinetics of Release

The equations describing the kinetics of release for bioactive compounds (GABA and polyphenols) include the following:

$$\mathrm{Zero}\text{-}\mathrm{order}\;\mathrm{equation}:\;Q_{t}=Q_{o}-K_{o}\times t$$

$$\mathrm{First}\text{-}\mathrm{order}\;\mathrm{equation}:\;Q_{t}=Q_{o}\times e^{-kt}$$

$$\mathrm{Higuchi}\;\mathrm{equation}:\;Q_{t}=K_{H}\times \sqrt{t}$$

$$\mathrm{Korsmeyer}\text{–}\mathrm{Peppas}\;\mathrm{equation}:\;Q_{t}=K_{M}\times t^{n}$$

where Q_t_ is the released percentage of GABA and TPC at time t (%); Q_o_ is the initial percentage of GABA and TPC (%); K_o_, K, K_H_, and K_M_ are the release constants for the zero-order, first-order, Higuchi, and Korsmeyer–Peppas equations, respectively; and *n* is the release exponent in the Korsmeyer–Peppas equation.

The best-fit kinetic model and release mechanism are selected based on the values of the determination coefficient (R^2^) and the release exponent (*n*) corresponding to each equation.

#### 2.5.10. Degradation Kinetics of Bioactive Compounds under Various Storage Conditions

The degradation of bioactive compounds (GABA and TPC) in encapsulated powder was calculated using a first-order kinetic equation as follows: lnC = lnC_o_ − k(t).

Here, C represents the concentration of bioactive compounds at time t; C_o_ is the initial concentration; k is the rate constant of degradation (day^−1^), calculated from the slope of the natural logarithm plot of C/C_o_ against time; and t is the storage time (days).

The half-life of degradation (t_1/2_) was determined at a specific temperature using the following equation: t_1/2_ = ln2/k.

The activation energy (Ea, kcal/mol) was calculated using the Arrhenius equation, as follows: k = Ax e^(−Ea/RT)^, where A is the frequency factor, R is the ideal gas constant (1.987 kcal/mol), and T is the absolute temperature (K).

#### 2.5.11. Equilibrium Moisture Content Determination

The moisture content (MC) equilibrium was determined using the BET (Brunauer Emmett Teller) and GAB (Guggenheim Anderson de Boer) equations as follows [20]:

$$\mathrm{BET}:\;MC=\frac{Aw}{\left(1-Aw\right)MC}=\frac{1}{M_{o}C}+\frac{\left(C-1\right)}{M_{o}C}Aw$$

$$\mathrm{GAB}:\;MC=\frac{M_{o}C_{o}K_{G}Aw}{\left(1-K_{G}Aw)(1-K_{G}Aw+C_{G}K_{G}Aw\right)}$$

where MC is the moisture content of the encapsulated powder, expressed as g/100 g dw; M_o_ is the grams of water equivalent to a monolayer adsorbed on 100 g dry substance; Aw is the water activity at moisture content MC; C is the BET constant; and C_o_, K_G_, and C_G_ are constants of the GAB equation.

### 2.6. Statistical Analysis

Optimization experimental data were analyzed using JMP version 13.0 (SAS Institute Inc., Cary, NC, USA). Model fitness was assessed by evaluating lack of fits, determination coefficients (R^2^), and Fisher’s F-test values obtained from analysis of variance (ANOVA). Statistical significance testing was based on the total sum of squares with 95% confidence. JMP version 13.0 was used to create second-degree polynomial models, three-dimensional (3D) surface plots, and two-dimensional (2D) contour plots for analyzing dependent variables. Model fitness was evaluated based on determination coefficients (R^2^), lack of fits, and Fisher’s F-test values obtained from analysis of variance (ANOVA). The second-degree polynomial equation was used to represent the relationship between the dependent variable (EE and EY in terms of GABA and TPC) as a function of independent variables. The equation is expressed as follows:

$$Y_{i}=a_{o}+a_{1}X_{1}+a_{2}X_{2}+a_{3}X_{3}+a_{11}X_{1}^{2}+a_{22}X_{2}^{2}+a_{33}X_{3}^{2}+a_{12}X_{1}X_{2}+a_{13}X_{1}X_{3}+a_{23}X_{2}X_{3}$$

where Y_i_ represents the dependent variables, a_o_ is the constant, a_i_, a_ii_, a_ij_ are the linear, quadratic, and interaction coefficients, respectively. X_i_ and X_j_ are the levels of the independent variables.

## 3. Results and Discussion

### 3.1. Fitting the Models

Based on the results of the single-factor preliminary trials, the optimal ranges of the investigated factors were determined. These factors include the ratio between two types of wall materials (GA:WP) (40:60, 50:50, and 60:40, *v*/*v*), the ratio of germinated mung bean extract to the wall material (20:100, 30:100, and 40:100, *v*/*v*), and the concentration of the wall material (15%, 20%, and 25%). The impact of the encapsulation formulations on the encapsulation efficiency and encapsulation yield in terms of GABA and total polyphenols (TPC) is presented in Table 2.

Table 3 shows the linear, quadratic, and interaction effects of the ratio between two wall materials (X_1_), the ratio of the extract to wall material (X_2_), and the wall material concentration (X_3_) on each dependent variables (Yi) of encapsulated powder. The estimated regression coefficients for Yi, accompanied by their respective R^2^ values, regression *p* values, and lack of fit *p* values, are presented in Table 3. ANOVA results, as depicted in Table 3, affirm the significance of the response surface models, and discernment reveals no significance in lack of fit (*p* > 0.05) within each model. The evaluation of the absolute t ratio and *p* values suggests a more pronounced effect on the associated variables [21]. The significance of the coefficients in the response surface models is detailed in Table 3. Hence, the models, characterized by high coefficients of determination (R^2^ ranging from 0.984 to 0.995), suffice to elucidate the impact of the examined independent variables on Yi. This implies that over 98% of the response variables, including EE-GABA, EE-TPC, EY-GABA, and EY-TPC, can be precisely explicated as functions of the investigated the ratio between two wall materials, the ratio of the extract to wall material, and the wall material concentration.

To generate the equations, only the significant regression coefficients (Table 3) were taken into account. The final equations in terms of the three independent variables, the ratio between two wall materials (X_1_), the ratio of the extract to wall material (X_2_), and the wall material concentration (X_3_) for encapsulation efficiency and encapsulation yield in terms of GABA and TPC to obtain the predicted values in Table 2 were as follows:

$$\mathrm{EE}\text{-}\mathrm{GABA}={43.03+1.48X}_{1}+1.46X_{3}-0.016X_{1}^{2}-0.008X_{2}^{2}-0.041X_{3}^{2}+0.005X_{1}X_{2}$$

$$\mathrm{EE}\text{-}\mathrm{TPC}=-85.55+2.36X_{1}-1.73X_{2}+12.5X_{3}-0.03X_{1}^{2}-0.28X_{3}^{2}+0.05X_{1}X_{2}$$

$$\mathrm{EY}\text{-}\mathrm{GABA}={-100.29+3.91X}_{1}+2.28X_{2}+4.74X_{3}+0.03X_{1}^{2}-0.04X_{2}^{2}-0.1X_{3}^{2}+0.02X_{1}X_{2}-0.03X_{1}X_{3}+0.04X_{2}X_{3}$$

$$\mathrm{EY}\text{-}\mathrm{TPC}={-61.11+2.45X}_{1}+1.96X_{2}+4.48X_{3}-0.02X_{1}^{2}-0.02X_{2}^{2}-0.11X_{3}^{2}-0.02X_{1}X_{2}$$

The quadratic models, which elaborate on the encapsulation efficiency and encapsulation yield of the formulation, are thoroughly examined in the subsequent sections (refer to Section 3.2 and Section 3.3). Specifically, the aim is to optimize the encapsulated GABA and TPC powder, deeming it an optimal product when the criteria for the optimization process lead to maximizing encapsulation efficiency and encapsulation yields for both GABA and TPC. The validation of these models is undertaken in Section 3.4.

### 3.2. Encapsulation Efficiency

Table 3 illustrates the influence of three independent variables on encapsulation efficiency (EE) in terms of GABA and TPC, as indicated by the coefficients of the quadratic model. Regarding the EE of GABA (EE-GABA), all regression coefficients significantly affected the EE-GABA. However, the linear term of X_2_ and interaction terms of X_1_X_3_ and X_2_X_3_ were deemed not significant (*p* > 0.05), indicating an absence of interaction between the ratio between two wall materials and the wall material concentration, as well as between the ratio of the extract to wall material and the wall material concentration with respect to the EE-GABA. The low surface GABA content in the microcapsules, ranging from 1.9 to 5.9%, led to an elevated EE-GABA. The EE-GABA exhibited variations from 98.1 to 94.1%. In the case of encapsulation efficiency for TPC (EE-TPC), most of the terms of independent variables significantly impacted the EE-TPC, except for quadratic terms of X_2_ and interaction terms of X_1_X_3_ and X_2_X_3_ (*p* > 0.05). The interaction terms for EE-TPC were found to be analogous to those observed for EE-GABA. However, the higher surface content of TPC in the encapsulated powder, ranging from 22.5 to 35.8%, resulted in lower encapsulation efficiency compared to the EE-GABA. In this study, it is evident that the EE-GABA was much higher than the EE-TPC due to the distinct properties of the core materials. However, the findings align with the literature data reporting that high encapsulation efficiency of different core materials using the freeze-drying method could be obtained [22,23].

**Figure 1:**
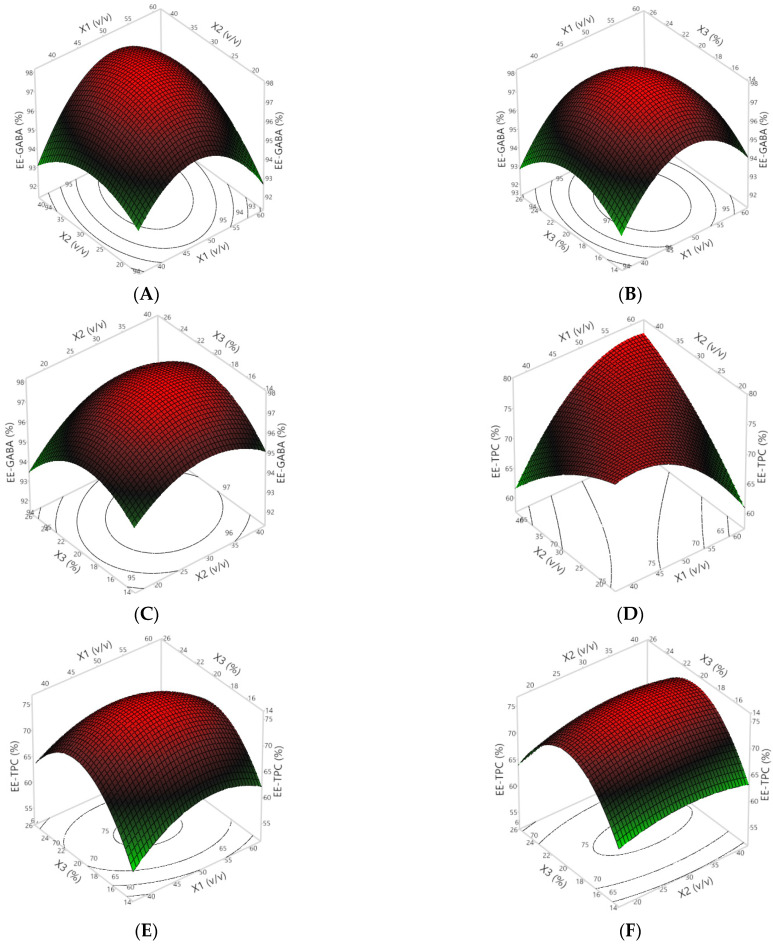
The 3D response and 2D contour plots of the EEs for GABA (**A**–**C**) and TPC (**D**–**F**) affected by the ratio between two wall materials (X_1_), the ratio of the extract to wall materials (X_2_), and the wall material concentration (X_3_).

To further elucidate optimal levels of the independent variables, 3D surface and 2D contour plots (Figure 1) were generated. These plots serve to visualize the relationship between the independent and response variables, with the response surface plot depicting this relationship, while the contour plot aids in grasping the shape of the response surface. Consequently, the utilization of these plots is imperative for evaluating the model fits [24]. Figure 1 shows the effect of the ratio between two wall materials, the ratio of the extract to wall materials, and the wall material concentration on EE-GABA and EE-TPC. It is interesting to note that EE-GABA and EE-TPC were significantly impacted by quadratic terms. In general, it can be seen that an increase in EE-GABA (Figure 1A–C) and EE-TPC (Figure 1D–F) could be achieved by increasing the two independent variables at a constant one variable. Therefore, it can be easily determined the optimal conditions of the formulation lead to the maximal encapsulation efficiency.

As discussed in Section 1, to achieve higher encapsulation efficiency, it is desirable to utilize combinations of various wall materials, such as WP and GA. This is because a singular wall material cannot fulfill all the required properties or characteristics within the microcapsule. Many studies have reported that the use of different blends of wall material significantly enhances the encapsulation efficiency of various bioactive compounds such as polyphenols from grape seed extract [25], catechins from green tea [26], and chlorophylls from spirulina [5].

In this study, a blend of GA and WP at the same ratio significantly improved the EEs (Table 2 and Figure 1). This improvement may be attributed to the specific components of GA and WP that make them valuable in the synthesis of microcapsules. GA, characterized by its branched heteropolysaccharides linked with proteins, serves as an outstanding film-forming agent, thereby enhancing the encapsulation of the encapsulated molecule. WP can undergo self-assembly or co-assembly into superstructures, facilitating the encapsulation and conveyance of diverse small molecules. Both GA and WP can function as surfactants, facilitating the creation and preservation of stable emulsions containing bioactive compounds. Furthermore, they have the capability to establish covalent or electrostatic complexes with targeted molecules, thereby immobilizing bioactive compounds through the formation of stable solutions [27].

There is a widely acknowledged consensus in the literature that higher core loads typically result in lower encapsulation efficiency, leading to diminished retention and a higher concentration of core material at the microcapsule surface. This occurrence is attributed to the inadequate amount of wall materials used, resulting in an insufficient encapsulation of the core material. As a consequence, core materials that are not effectively encapsulated within the wall matrix become more susceptible to degradation from heat and/or oxidation, ultimately causing a decline in encapsulation efficiency. In simpler terms, a higher concentration of core materials may contribute to an increased presence of these materials on the powder surface, subsequently elevating the surface levels of GABA and TPC in the microcapsules.

The concentration of the wall material showed a positive impact on the encapsulation efficiencies concerning the retention of GABA and TPC, where both lower and higher concentrations resulted in decreased encapsulation efficiency (Figure 1). This phenomenon can be explained by the lower concentrations of the wall materials and higher ratios of the extract solution to the wall material solution, which may have led to insufficient quantities of wall materials available to fully encapsulate the bioactive compounds. This inadequacy could result in some degradation during the preparation and freeze-drying steps. The interaction effect between the concentration of the wall materials and the ratio of the extract solution to the wall material solution on the EE-GABA (*p* < 0.05) and the EE-TPC (*p* < 0.001) is evidenced in Table 3. Furthermore, it is important to note that a high concentration of wall material is unexpected, as it results in low amounts of GABA and TPC in the microcapsules. Therefore, optimizing the concentration of wall materials to achieve the highest encapsulation efficiency is essential.

### 3.3. Encapsulation Yield

**Figure 2:**
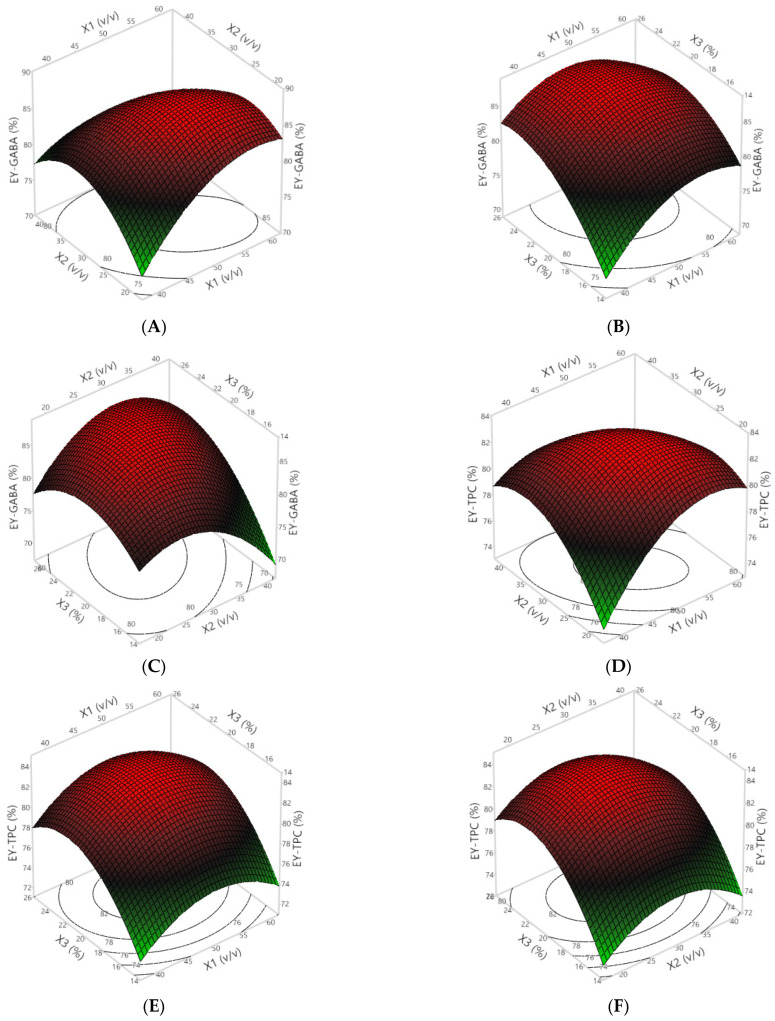
The 3D response and 2D contour plots of the EYs for GABA (**A**–**C**) and TPC (**D**–**F**) affected by the ratio between two wall materials (X_1_), the ratio of the extract to wall material (X_2_), and the wall material concentration (X_3_).

In addition to EE, achieving a higher encapsulation yield (EY) stands as a crucial objective in any encapsulation process, including freeze-drying, representing one of its major independent variables. A low yield can render encapsulation uneconomical, making it potentially not worthwhile. Therefore, understanding the effects of key independent variables in the system is essential for optimizing the yield. The EY of the encapsulated powders, formulated with varying ratios between wall materials (X_1_), ratios of the extract to wall materials (X_2_), and wall material concentrations (X_3_), is presented in Table 2 and Table 3 and Figure 2. Generally, the results indicate a notably higher EY in terms of GABA with increasing X_1_, X_2_, and X_3_, as indicated by the significant values of all regression coefficients (*p* < 0.05). A similar trend was observed for the effect of X_1_, X_2_, and X_3_ on the EE-TPC; however, there were no statistical significances in the interaction between X_1_ and X_3_, and X_2_ and X_3_ (*p* > 0.05).

In this study, the encapsulation yield in terms of GABA and TPC ranged from 73.63 to 87.31 and 75.61 to 83.14, respectively (Table 2). Previously, similar EY values were reported for different materials including encapsulated Pulicaria jaubertii extract (83.16–87.95%) [28], probiotic starter culture (86.04–88.10%) [29], and Citrus aurantium essential oil (77.61–90.22%) [30]. According to Al-Maqtari et al. [28], the combination of WP and GA to create the wall materials proved to enhance the encapsulation yield in the prepared microencapsulated Pulicaria jaubertii extract compared to microcapsules prepared with GA alone. This is because wall materials containing WP and GA create a coating with diminished internal impact and constrained convection heat transfer. This leads to minimal bioactive compound loss through volatilization or surface diffusion during subsequent operations [28,31].

In general, the higher encapsulation yield obtained is one of the most advantageous aspects of the lyophilization process, along with the high retention of bioactive compounds. In this study, the encapsulation yields and encapsulation efficiency for TPC can be further improved in future research. Since this current study focuses on optimizing the encapsulation formulation, there is a need to determine the optimal parameters of the entire freeze-drying process parameters, such as the type and duration of homogenization, freezing rate, and freezing temperature, among others, to improve the encapsulation yields. Additionally, several studies have indicated that the encapsulation efficiency and encapsulation yield through the freeze-drying method is significantly higher than other drying methods such as spray-drying, which is widely applied in the food industry [3,32]. This is attributed to the use of lower drying temperatures, which is particularly suitable for bioactive compounds such as GABA and polyphenols sensitive to high temperatures.

### 3.4. Overall Optimization and Model Validation

It is widely recognized that employing response surface methodology with a Box–Behnken design represents one of the most efficient approaches for assessing the correlation between the response and independent variables, subsequently facilitating the optimization of processing conditions and formulations. This method enables the assessment of the impact of multiple parameters and their interactions on output variables with a reduced number of trials. Numerous review papers extensively analyze response surface methodology, outlining its advantages and drawbacks, as reported by various authors [24,33]. Furthermore, numerous studies affirm the successful utilization of response surface methodology in optimizing formulations and various processes [34,35,36]. It is noteworthy that this technique is not only swifter but also more cost-effective compared to alternative approaches for process optimization.

**Figure 3:**
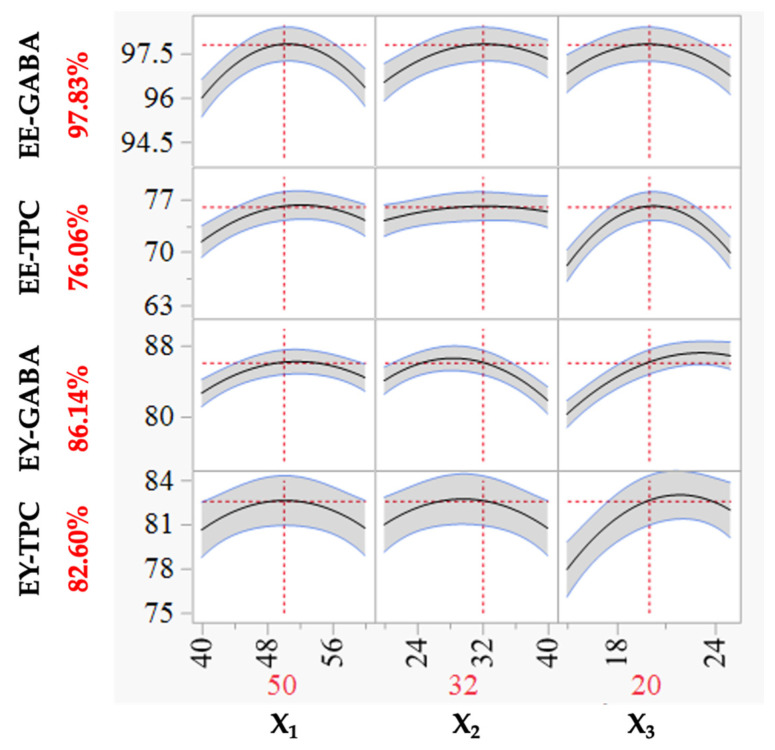
Prediction profilers of EEs and EYs for GABA and TPC as a function of the ratio between two wall materials (X_1_), the ratio of the extract to wall material (X_2_), and the wall material concentration (X_3_).

The objective of this research was to determine the optimal encapsulation formulation; thus, an optimization method was implemented using JMP software version 13.0. The optimized model results in the highest EE and EY in terms of GABA and TPC. The prediction profilers illustrated in Figure 3 demonstrate that the optimal formulation, meeting the criteria for the response variables, can be determined within the explored range for the encapsulated powder by adjusting the position of the vertical dot lines to the left or right. The corresponding horizontal dot lines indicate the achieved values of the response variable. The findings in Figure 3 have identified the optimized encapsulation formulation, comprising a GA: WP ratio (X_1_, *v*/*v*) of 50:50, an extract: wall material ratio (X_2_, *v*/*v*) of 30:100, and a wall material concentration (X_3_) of 20%. With this optimized encapsulation formulation, the EE-GABA (97.83%), EE-TPC (76.06%), EY-GABA (86.14%), and EY-TPC (82.60%) have been optimized.

Moreover, conducting a comparison between predicted and measured response values serves as a means to assess the accuracy of the model describing the studied encapsulation process. Consequently, a validation experiment was conducted in triplicate under optimal conditions. The results indicated that the measured dependent variables, including the EE-GABA, EE-TPC, EY-GABA, and EY-TPC were found to be 98.87%, 79.52%, 88.60%, and 85.40%, respectively. Statistical analysis reveals no significant disparity between the predicted and measured values for the value of EEs and EYs. Therefore, a second-degree polynomial equation can be employed to predict the encapsulation efficiencies and encapsulation yields of GABA and TPC. In simpler terms, the response surface optimization method for encapsulation conditions of mung bean extract was of high practical value.

### 3.5. Particle Morphology and Size Distribution

**Figure 4:**
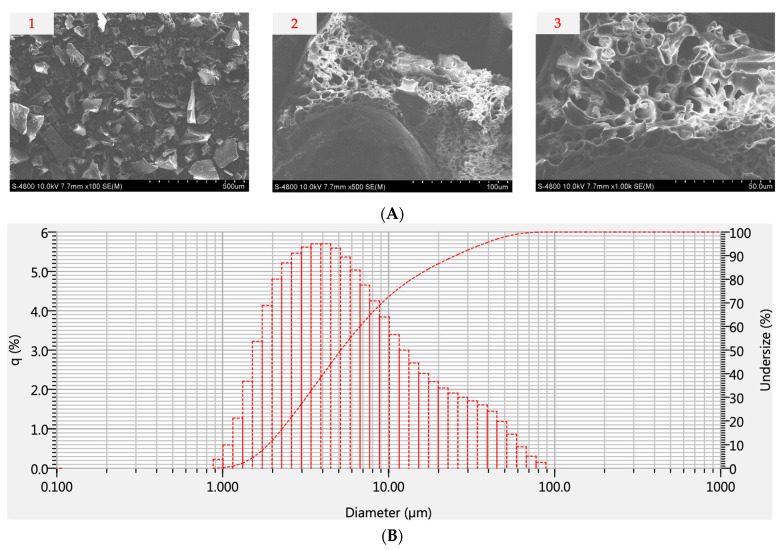
SEM images at different magnifications of (**A1**) ×100, (**A2**) ×500, and (**A3**) ×1000 and the particle size distribution of the encapsulated powder (**B**).

The surface morphology is a key factor influencing the quality of the encapsulated microencapsulate particles. The surface structure of these particles not only impacts the particles themselves but is also closely tied to physicochemical properties, such as fluidity and dispersibility during storage. As the stability of the encapsulated powder is important, there is a need to investigate the degradation of bioactive compounds in the powder under different storage conditions (refer to Section 3.7). Concurrently, the particle size distribution plays a crucial role in the blending with liquids during the utilization or processing of the powder. The structural analysis and particle size distribution of the encapsulated powder are illustrated in Figure 4. The result shows that the images of the freeze-dried encapsulated powder exhibit irregular shapes resembling shattered glass, including concavities and dents. The structure of all particles (Figure 4A) is uneven, brittle, porous, and cake-like, akin to the findings reported by various authors [3,37]. It is well-known that the porous characteristics result from the formation of ice crystals during freezing, which subsequently undergo sublimation during the freeze-drying process. In addition, it is evident that there was no agglomeration or fusion of the microparticles.

SEM images (Figure 4A) reveal the formation of indistinct glass-like particles on the surface of ultra-small capsules, indicating the presence of the encapsulating material on the surface, which acts as a protective barrier for the encapsulated molecules, shielding them from direct exposure to heat and oxygen. Kuck and Noreña [38] observed that the development of a highly porous encapsulated powder results from the formation of crystals within the material during encapsulation, effectively preventing structural contraction and collapse, leading to a substantial retention of mass. Correspondingly, Mahdavee Khazaei et al. [39] reported similar traits in encapsulated powder with maltodextrin. The highly dispersed ultra-small capsules contribute to improved phenolics solubility in food. Çam et al. [40] also noted that the mass of the encapsulating material is adequate to ensure the prolonged stability of the core material (phenolics). Ballesteros et al. [41] further endorsed the freeze-drying method as the most efficacious for phenolic encapsulation. Thus, SEM images of the encapsulated powder sample depict a matrix-like structure, irregular shape, and numerous porous voids inside, consistent with findings in studies on encapsulation particles using the freeze-drying method published by various researchers.

According to Figure 4B, roughly 75% of the encapsulated powder passes through a 10 μm sieve, 90% passes through a 25 μm sieve, and 100% passes through a 90 μm sieve. This feature is particularly beneficial for real-world manufacturing, as it aligns with the prevalent use of filter sizes ranging between 100 and 150 μm. Furthermore, it is noteworthy that the encapsulated powder demonstrates a relatively even distribution, with an average size of approximately 10 μm, suggesting its potential for stable and uniform dispersion in a solution.

### 3.6. Release Kinetics of Bioactive Compounds under Various Conditions

**Figure 5:**
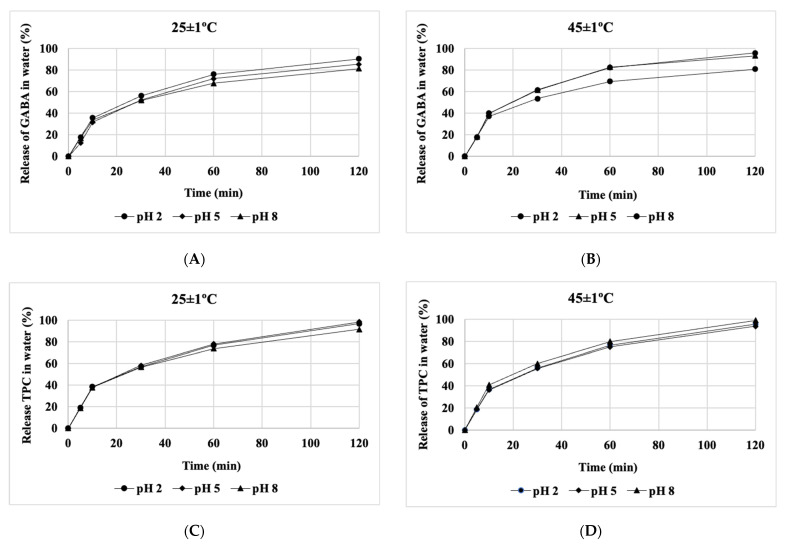
The influence of temperature on the release of GABA and TPC at different pH levels in water. (**A**–**D**) depict the release of GABA and TPC in water at different temperatures of 25 and 45 °C, respectively.

**Figure 6:**
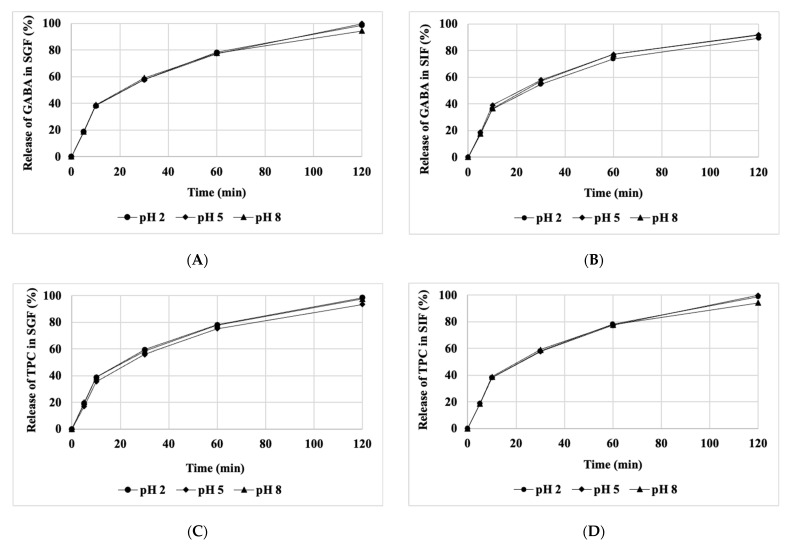
The influence of pH on the release of GABA and TPC in SGF and SIF at 37 °C. (**A**–**D**) depict the release of GABA and TPC in SGF and SIF, respectively.

The release of GABA and polyphenols from lyophilized powder has been investigated under different temperature conditions, varying pH levels, and in different environments. The powder samples were introduced into aqueous solutions, SGF, and SIF with varying pH values (2, 5, and 8). GABA and polyphenol concentrations for each sample were measured at different temperatures over a total duration of 120 min. The cumulative release of GABA and polyphenols in the aqueous environment at different temperatures and pH levels is illustrated in Figure 5, while the release of GABA and total polyphenolic content (TPC) in SGF and SIF environments at varying pH levels is presented in Figure 6. Overall, the release rates of GABA and polyphenols under the investigated conditions (pH, temperature, and environment) increased over time, reaching 81.5% and 99.8%, respectively, after 120 min. However, the release rates of GABA and polyphenols exhibited variations under different experimental conditions.

According to the results presented in Figure 5 and Figure 6, at a temperature of 25 °C, the release rate of TPC content in all three environments, including water, SGF, and SIF, is faster than the release rate of GABA. However, when increasing the temperature from 37 to 45 °C, the difference in the release rates of GABA and TPC becomes insignificant. At the three temperature levels investigated, the release rates of both GABA and TPC at different pH levels show little variation. Nevertheless, the release rates of GABA and TPC in the aqueous environment at 45 °C are higher than those at 37 °C (Figure 5). Here, the impact of higher temperature accelerates the release rate compared to lower temperatures.

In general, in all three environments, the rapid release of GABA and polyphenols at low pH levels (pH 2 and 5) was faster compared to pH 8. Changes in temperature and pH can induce structural alterations within the matrix, thereby accelerating the release of bioactive compounds. These findings align with a study on the release of polyphenols from peppermint leaves conducted by Rout et al. [4].

Table 4 and Table 5 present the regression coefficients of zero-order, first-order, Higuchi model, and Korsmeyer–Peppas model equations for the release of GABA and TPC under different environmental conditions. These models are employed to predict the release mechanisms of GABA and polyphenols under various conditions. Overall, the results indicate that the Korsmeyer–Peppas and Higuchi models provide the most suitable fit and can be utilized to predict the release rates of GABA and polyphenols, given their high R^2^ coefficients.

In the four models used for investigation, the overall release rate can be explained by both the Korsmeyer–Peppas and Higuchi models. According to Flores and Kong [42], there might be cases where the release process of bioactive compounds like polyphenols is adequately described by two different mathematical models simultaneously. The Higuchi coefficient (K_H_) is meaningful as it can depict a pseudo-steady state, depending on the release of phenolic compounds from lyophilized powder. Additionally, the value of n in the Korsmeyer–Peppas model predicts the release mechanism of GABA and polyphenols under different temperature and pH conditions. Fickian diffusion occurs when *n* ≤ 0.43, suggesting that the relaxation time of the matrix wall is greater than the characteristic permeation time of the solution. Here, the release process is entirely through permeation. When the value of *n* is greater than 0.43, the release mechanism of GABA and polyphenols does not follow Fickian diffusion, meaning the release mechanism may occur due to a combination of permeation and erosion [4,42]. In this study, at pH 5, the release mechanism adheres to both permeation and erosion laws.

In this study, the insights gained from the release kinetics of bioactive compounds from the encapsulated matrix prove valuable for predicting the release rate of GABA and TPC under various simulated environmental conditions. The results offer a straightforward and practical understanding of bioactive compound release. Moreover, the established models are simple yet detailed, providing insights into the release mechanism and emphasizing influential factors such as medium, temperature, and pH. However, a notable current limitation in controlled release mathematical modeling is the reliance on studies conducted in simulated food systems. Real food products, being intricate systems, lack well-defined underlying mechanisms. While the developed models aid in selecting suitable carriers with appropriate release rates for industrial food product development, it is strongly recommended to investigate release mechanisms in real food products. Moreover, future research is expected to shift its primary focus to assessing the bioavailability of released bioactive agents within the human body. The integration of novel computational techniques is anticipated to enhance the resolution of complex release models [43].

### 3.7. Degradation Kinetics of Bioactive Compounds under Various Storage Conditions

#### 3.7.1. The Effects of Temperature

**Figure 7:**
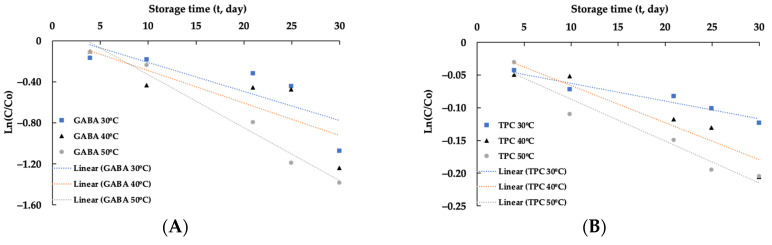
The degradation of GABA (**A**) and TPC (**B**) at different storage temperatures.

The reduction in GABA and TPC in encapsulated samples during storage aligns with a first-order decay equation (Figure 7). Table 6 presents the kinetic parameters of the GABA and TPC decay process in encapsulated powder at various storage temperatures (30, 40, and 50 °C). The results in Table 4 indicate that the degradation rate of GABA is significantly higher than that of TPC, ranging from 5 to 10 times at different storage temperatures. Examining each bioactive compound individually, such as GABA, the degradation rate was higher when the encapsulated powder was stored at elevated temperatures. Similar trends are observed in the degradation rate of TPC with an increase in storage temperature from 30 °C to 50 °C. The differential degradation rates between GABA and TPC in the encapsulated powder may stem from the distinct molecular structures of the two compounds and their varied interactions with the protein-polysaccharide matrix of the encapsulating material during storage. In-depth research is needed to comprehend the diverse interactions among bioactive compounds and the surrounding matrix, thereby understanding alterations in the physical and chemical properties of encapsulated particles. Such insights can inform the development of release mechanisms and applications in the food industry. In this study, an investigation into the rate and prediction of the release mechanism of encapsulated powder containing GABA and TPC under different pH and temperature conditions is presented in Section 3.5.

The activation energies for the decay processes of GABA (5.85 kcal) and TPC (10.72 kcal) at different storage temperatures are presented in Table 6. Notably, the activation energy of TPC is 1.8 times higher than that of GABA. Generally, the activation energy of a chemical reaction represents the minimum energy required to initiate the reaction. In this context, the disparity in activation energy between GABA and TPC may be attributed to differences in chemical structure and interactions with the protein and polysaccharide matrix of these compounds.

Based on the degradation time parameters (Table 6), it is straightforward to predict the storage duration for the encapsulated powder. According to Table 6, it can be predicted that the half-life of the encapsulated powder could be achieved to be approximately 2 months or 15 months at 20 °C based on the retention of GABA or TPC, respectively. It is evident that a longer shelf life for the encapsulated powder can be achieved at lower storage temperatures. Therefore, the encapsulated powder should ideally be stored at lower temperatures to extend its storage duration.

#### 3.7.2. The Effects of Air Relative Humidity

**Figure 8:**
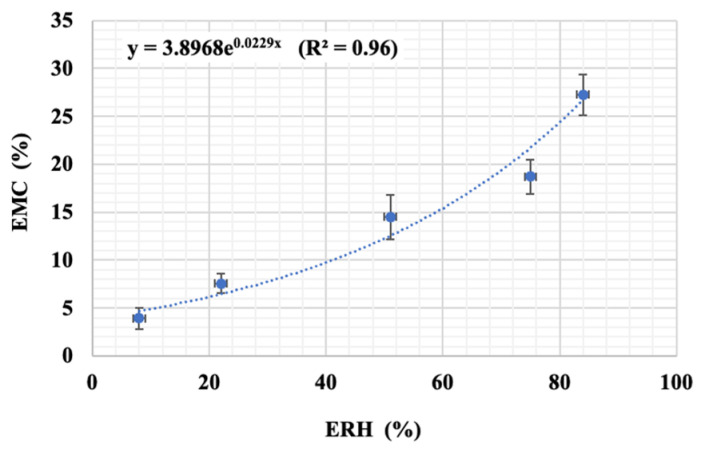
The effect of equilibrium relative humidity (ERH) on equilibrium moisture content (EMC) of the encapsulated powder under ambient temperature.

The impact of relative air humidity on sprouted green pea extract encapsulated powder was determined at ambient temperature under five distinct relative humidity conditions. The time for the samples to reach equilibrium mass status was determined. Figure 8 illustrates the relationship between the equilibrium moisture content (EMC_dw_, %) and equilibrium relative humidity (ERH, %) of the encapsulated powder samples. This curve is utilized to predict the storage duration of the product concerning stability in terms of physical, biochemical, and bacterial aspects when establishing packaging and storage conditions [44,45]. Typically, the EMC values at a constant temperature increase with the rise in ERH. This aligns with the perspective of several authors suggesting that moisture graphs for dry foods often exhibit a curve. In the low ERH range (8–51%), the encapsulated powder samples experience a minimal increase in EMC. The moisture content and activity of samples within this range are low, indicating a very small amount of free water in the powder. Consequently, the samples may exhibit enhanced stability in terms of physical, chemical, and biological aspects. However, introducing food into this ERH range through the drying process is challenging and costly. Conversely, physical reactions in the samples occur easily and rapidly at ERH values exceeding 75% due to high moisture absorption, conditions that promote chemical, biological, and bacterial degradation processes. In the ERH range from 51% to 75%, the EMC values show minimal variations, indicating stable water absorption without additional increments.

According to the BET equation, the monolayer moisture content (M_o_, % on a dry basis) calculated for the encapsulated powder sample is 4.70% (R^2^ = 0.94). However, the M_o_ value (9.95%) with an R^2^ = 0.87, when calculated using the GAB equation, is significantly higher. This outcome indicates that the BET equation is more accurate in predicting M_o_, as the M_o_ values generated exhibit a higher R^2^ value. Overall, the M_o_ value of a dry food product is considered the safest moisture content for stable storage. This is because chemical, physical, and biological reactions are slowed down at moisture levels below M_o_, while reactions occur at higher moisture levels due to increased Maillard browning, as well as enzymatic and bacterial activities. Therefore, dry powder samples should ideally have an initial moisture content equal to or lower than the M_o_ value [46]. In this study, the moisture content of the encapsulated germinated mung bean extract powder was found to be less than 4%, indicating the potential for long-term storage.

**Figure 9:**
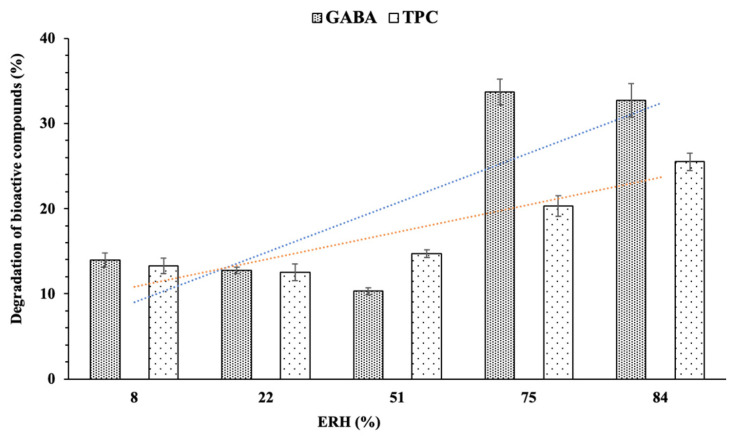
The influence of different equilibrium relative humidities (ERH) on the content of GABA and TPC.

The influence of relative humidity on the loss of bioactive compounds is depicted in Figure 9. The results indicate minimal losses in GABA and polyphenols (<15%) at relative humidity levels below 51%. However, losses significantly increase (over 30% for GABA and 25% for TPC) as the relative humidity rises to 84%. Exposure to air and the potential destruction of the encapsulated particle structure at high air humidity may contribute to the substantial reduction in GABA and TPC content in the powder sample. Furthermore, at such elevated air humidity levels, water exists in a free state and is likely to promote undesired chemical, biological, and bacterial reactions [47]. In this study, the encapsulated powder exhibited low moisture content (<4%) and low water activity (<0.4), indicating a high level of stability.

The kinetics involved in storing bioactive compounds under diverse conditions, including variables such as temperature and humidity, within the realm of food storage, play a pivotal role in establishing the optimal storage conditions for food powders through the assessment of degradation rates. This necessitates a comprehensive understanding of how factors like temperature and humidity levels impact the stability of the powder over time. Additionally, manufacturers can forecast the shelf life of food powders by discerning degradation rates and half-life. This information is crucial for tasks such as setting expiration dates, efficient inventory management, and ensuring consumers receive products of the desired quality (such as GABA and TPC) and safety.

As a result, this study makes a substantial contribution to the development of effective preservation and storage strategies, ultimately safeguarding the safety, quality, and durability of food products. This insight is of paramount importance for both producers and consumers in maintaining a reliable and secure food supply. Given that the shelf life of encapsulated powder can be influenced by various factors, including exposure to oxygen, light, and packaging, among others, further investigations into practical storage studies should be conducted. In conclusion, optimizing storage conditions can significantly extend the shelf life of the product.

## 4. Conclusions

The optimization of the freeze-drying formulation for encapsulating an aqueous germinated mung bean extract was successfully achieved through the utilization of response surface methodology with Box–Behnken design. Second-order polynomial models were developed and demonstrated statistical adequacy in describing and predicting various dependent variables, including the EE-GABA (R^2^ = 0.984), EE-TPC (R^2^ = 0.995), EY-GABA (R^2^ = 0.992), and EY-TPC (R^2^ = 0.995). Utilizing these established models, the optimal formulation was predicted and subsequently validated, including the ratio of GA to WP (X_1_), the ratio of the extract to wall material (X_2_), and the wall material concentration (X_3_). The predicted values for the EE-GABA, EE-TPC, EY-GABA, and EY-TPC were 97.83%, 76.06%, 86.14%, and 82.60%, respectively.

The study’s findings on the release kinetics of encapsulated particles under varied conditions (temperature, pH, and time) highlighted the Korsmeyer–Peppas and Higuchi models as particularly fitting, given their high R^2^ coefficients. These models can effectively predict the release rates and mechanisms of GABA and polyphenols. Additionally, the stability of the encapsulated powder was significantly affected by fluctuations in storage temperatures (40, 50, and 60 °C) and relative humidities (8.2–84.5%). Degradation rates of the encapsulated microparticles were determined, providing insights into predicting the powder’s shelf life under specific temperature conditions. The moisture equilibrium of the encapsulated powder, calculated using the BET model, was established at 4.70% (R^2^ = 0.94). With a moisture content below 4%, the encapsulated powder is considered highly suitable, indicative of long-term stability. In conclusion, the encapsulated germinated mung bean extract powder is highly stable and can be successfully incorporated into food products and/ or used as functional ingredients. The recommendation is to further explore the practical applications of the encapsulated powder in these products. This exploration should encompass conducting kinetics studies on the degradation and release of GABA and TPC.

## Figures and Tables

**Table 1:** Coded and uncoded levels for different independent variables.

Independent Variable	Coded Variable Level		
	−1	0	1
The ratio between two wall materials (X_1_, *v*/*v*)	40:60	50:50	60:40
The ratio of the extract to wall material (X_2_, *v*/*v*)	20:100	30:100	40:100
The wall material concentration (X_3_, %)	15	20	25

**Table 2:** The experimental (Exp.) and predicted (Pred.) values for the encapsulation efficiency (EE) and encapsulation yield (EY) in terms of GABA and total polyphenols for the encapsulated powder obtained by different formulations.

Exp Run ^a^	Patterns	X_1_ (*v*/*v*)	X_2_ (*v*/*v*)	X_3_ (%)	EE-GABA (%)		EE-TPC (%)		EY-GABA (%)		EY-TPC (%)	
					Exp.	Pred.	Exp.	Pred.	Exp.	Pred.	Exp.	Pred.
1	0++	50	40	25	96.50	96.47	70.27	69.31	84.59	84.21	80.16	79.91
2	000	50	30	20	97.83	97.79	75.70	75.99	87.31	86.50	82.80	82.70
3	+−0	60	20	20	94.13	94.40	67.62	66.44	84.54	84.90	81.56	80.98
4	0−−	50	20	15	95.82	95.85	65.69	66.65	80.27	80.65	75.78	76.03
5	+0−	60	30	15	95.53	95.23	66.32	66.54	81.76	81.03	76.12	76.45
6	+0+	60	30	25	95.27	95.11	64.66	65.62	83.90	84.24	79.34	80.51
7	0−+	50	20	25	95.28	95.17	67.59	67.81	83.03	82.34	81.17	80.58
8	000	50	30	20	97.38	97.79	76.98	75.99	86.71	86.50	82.16	82.70
9	++0	60	40	20	96.13	96.32	77.52	77.52	78.33	78.37	78.53	77.61
10	−+0	40	40	20	95.33	95.06	65.56	66.74	80.41	80.05	79.40	79.98
11	000	50	30	20	98.15	97.79	75.29	75.99	85.49	86.50	83.14	82.70
12	−0−	40	30	15	94.98	95.14	64.23	63.27	76.16	75.83	76.81	75.64
13	−0+	40	30	25	94.61	94.91	67.69	67.47	83.47	84.20	80.15	79.82
14	0+−	50	40	15	96.02	96.14	67.40	67.18	73.63	74.32	75.61	76.20
15	−−0	40	20	20	95.56	95.37	75.19	75.80	78.02	77.98	76.19	77.11

^a^ Experiments were run in random order; **X_1_**: the ratio between two wall materials; **X_2_**: the ratio of the extract to wall material; and **X_3_**: the wall material concentration.

**Table 3:** The regression coefficients of the second-degree polynomial equation describe the influence of the encapsulation formulation on encapsulation efficiency (EE) and encapsulation yield (EY) in terms of GABA and TPC.

Regression Coefficients ^a^	EE-GABA (%)		EE-TPC (%)		EY-GABA (%)		EY-TPC (%)	
	Regression Coefficients	t Ratio	Regression Coefficients	t Ratio	Regression Coefficients	t Ratio	Regression Coefficients	t Ratio
a_o_	43.03 **	5.00	−85.55 *	−3.04	−100.29 **	−4.84	−61.11	−2.43
Linear								
a_1_	1.48 **	6.50	2.36 *	3.15	3.91 ***	7.12	2.45 *	3.66
a_2_	0.17	0.94	−1.73 *	−2.99	2.28 **	5.35	1.96 *	3.79
a_3_	1.46 *	3.63	12.50 ***	9.50	4.74 **	4.90	4.84 **	4.11
Quadratic								
a_11_	−0.016 ***	−8.05	−0.032 **	−4.86	−0.03 **	−5.29	−0.02 *	−3.21
a_22_	−0.008 **	−4.12	−0.012	−1.86	−0.04 ***	−7.20	−0.02 *	−3.09
a_33_	−0.041 **	−5.04	−0.28 ***	−10.42	−0.10 **	−5.18	−0.11 **	−4.44
Interaction								
a_12_	0.005 *	2.83	0.05 ***	7.75	−0.02 **	−4.53	−0.02 *	−2.7
a_13_	0.0005	0.14	−0.027	−1.98	−0.03 *	−2.72	−0.0006	−0.05
a_23_	0.0051	1.29	0.005	0.38	0.04 **	4.32	−0.004	−0.36
R^2^	0.984		0.995		0.992		0.995	
Regression *p* value	0.0061		0.0018		0.0011		0.0189	
*p*-value of lack of fit	0.517		0.266		0.513		0.110	

* *p* < 0.05, ** *p* < 0.01, *** *p* < 0.001. ^a^ a_o_ is a constant, a_i_, a_ii_, a_ij_ are the linear, quadratic, and interaction coefficients of the second-order polynomial equation, respectively.

**Table 4:** Regression coefficients of predictive models for the release rate of GABA and polyphenols at different pH and temperature conditions in water.

Release Conditions			Zero-Oder		First-Order		Higuchi Model		Korsmeyer–Peppas			Mechanisms
Bioactive Compounds	Temperature (°C)	pH	K_o_	R^2^	K	R^2^	K_H_	R^2^	K_M_	n	R^2^	
**GABA**	**25**	**2**	0.0358	0.8430	0.0131	0.7546	0.4646	0.9907	0.6160	0.4331	0.9940	Fickian
		**5**	0.0357	0.8635	0.0142	0.7170	0.4407	0.9908	0.4844	0.4777	0.9915	Non-Fickian
		**8**	0.0318	0.8329	0.0124	0.7502	0.4203	0.9890	0.5918	0.4188	0.9915	Fickian
	**45**	**2**	0.0360	0.7916	0.0130	0.6925	0.4870	0.9783	0.7294	0.4041	0.9859	Fickian
		**5**	0.0350	0.7746	0.0127	0.6978	0.4816	0.9743	0.7898	0.4923	0.9753	Non-Fickian
		**8**	0.0294	0.7704	0.0116	0.7010	0.4169	0.9746	0.7182	0.3705	0.9877	Fickian
**TPC**	**25**	**2**	0.0204	0.8757	0.0098	0.6204	0.1992	0.9899	0.2779	0.4210	0.9943	Fickian
		**5**	0.0207	0.8773	0.0100	0.6175	0.2022	0.9900	0.2761	0.4261	0.9940	Fickian
		**8**	0.0194	0.8644	0.0094	0.5889	0.1912	0.9859	0.2841	0.4053	0.9925	Fickian
	**45**	**2**	0.0201	0.8774	0.0098	0.6161	0.1967	0.9904	0.3249	0.3800	0.9934	Fickian
		**5**	0.0211	0.8943	0.0103	0.6542	0.2045	0.9952	0.2525	0.4501	0.9969	Non-Fickian
		**8**	0.0210	0.8653	0.0060	0.6372	0.2063	0.9871	0.3100	0.4034	0.9936	Fickian

**Table 5:** Regression coefficients of predictive models for the release rate of GABA and polyphenols at different pH levels in SGF and SIF.

Release Conditions		Zero-Oder		First-Order		Higuchi Model		Korsmeyer–Peppas			Mechanisms
Bioactive Compounds	pH	K_o_	R^2^	K	R^2^	K_H_	R^2^	K_M_	n	R^2^	
**GABA in SGF**	**2**	0.0368	0.8031	0.0127	0.7144	0.5020	0.9826	0.7736	0.3972	0.9906	Fickian
	**5**	0.0360	0.7813	0.0121	0.7009	0.5102	0.9780	0.8866	0.3684	0.9909	Fickian
	**8**	0.0360	0.8122	0.0125	0.7306	0.4898	0.9851	0.7560	0.3969	0.9928	Fickian
**GABA in SIF**	**2**	0.0332	0.8191	0.0125	0.7366	0.4445	0.9860	0.6476	0.4106	0.9920	Fickian
	**5**	0.0340	0.8011	0.0125	0.7169	0.4634	0.9819	0.7157	0.3967	0.9900	Fickian
	**8**	0.0344	0.8116	0.0128	0.7246	0.4592	0.9839	0.6634	0.4127	0.9899	Fickian
**TPC in SGF**	**2**	0.0207	0.8730	0.0099	0.6210	0.2031	0.9890	0.3181	0.3884	0.9936	Fickian
	**5**	0.0197	0.8779	0.0100	0.5809	0.1925	0.9891	0.2911	0.3982	0.9925	Fickian
	**8**	0.0205	0.8713	0.0097	0.6296	0.2015	0.9890	0.3316	0.3776	0.9938	Fickian
**TPC in SIF**	**2**	0.0155	0.8418	0.0100	0.6174	0.2022	0.9902	0.2739	0.4280	0.9939	Fickian
	**5**	0.0156	0.8509	0.0101	0.6257	0.2027	0.9917	0.2714	0.4302	0.9949	Fickian
	**8**	0.0147	0.8106	0.0096	0.5901	0.1982	0.9841	0.3006	0.4007	0.9911	Fickian

**Table 6:** Degradation kinetics of GABA and TPC in the encapsulated powder.

Kinetic Parameters	GABA			TPC		
	30 °C	40 °C	50 °C	30 °C	40 °C	50 °C
Degradation rate (k, day^−1^)	0.0284	0.0316	0.0517	0.0027	0.0057	0.0064
Half-life (t_1/2,_ day)	24.41	21.94	13.41	256.72	121.60	108.30
Determination coefficients (R^2^)	0.81	0.85	0.97	0.93	0.90	0.94
Activation energy (Ea, kcal)	5.85			10.72		

## Data Availability

Data is contained within the article.
